# Hormonal Contraceptive Use and Breast Cancer in Thai Women

**DOI:** 10.2188/jea.JE20130121

**Published:** 2014-05-05

**Authors:** Arisara Poosari, Supannee Promthet, Siriporn Kamsa-ard, Krittika Suwanrungruang, Jirapat Longkul, Surapon Wiangnon

**Affiliations:** 1 Department of Epidemiology, Faculty of Public Health, Khon Kaen University, Khon Kaen, Thailand; 2 Department of Biostatistics and Demography, Faculty of Public Health, Khon Kaen Universtity, Khon Kaen, Thailand; 3 Cancer Unit, Faculty of Medicine, Khon Kaen University, Khon Kaen, Thailand; 4 Department of Pediatrics, Faculty of Medicine, Khon Kaen University, Khon Kaen, Thailand

**Keywords:** hormonal contraceptive, breast cancer, Thai women

## Abstract

**Background:**

Breast cancer is the most common cancer in women worldwide. We investigated the association of hormonal contraceptive use and breast cancer in Thai women.

**Methods:**

A cohort study was conducted in Khon Kaen, Thailand. There were 70 cases of histologically confirmed breast cancer among 11 414 women aged 30 to 69 years who were recruited as participants in the cohort study during the period from 1990 through 2001. The study population was followed-up until December 31, 2011. To identify factors associated with incidence of breast cancer, hazard ratios (HRs) and 95% confidence intervals (CIs) were estimated using a Cox proportional hazards model.

**Results:**

The 11 414 women provided a total observation time of 157 200 person-years. Breast cancer risk among women with a history of hormonal contraceptive use was 1.31 times that of women without such a history, but the difference was not statistically significant (95% CI, 0.65–2.65). No type of hormonal contraceptive was associated with a significant increase in breast cancer risk as compared with women who had never used hormonal contraceptives (oral contraception: HR = 1.35, 95% CI, 0.65–2.78; injection contraception: HR = 1.25, 95% CI, 0.56–2.80), and there was no relationship between duration of hormonal contraceptive use and breast cancer.

**Conclusions:**

There was no association between hormonal contraceptive use and breast cancer; however, this finding should be viewed with caution due to the small number of cases.

## INTRODUCTION

Breast cancer is the most common cancer in women worldwide. Globally, breast cancer accounts for 23% of all cancers in women.^1^ The International Agency for Research on Cancer (IARC) estimated the incidences of 27 types of cancer in the world in 2008 and identified 1.38 million new cases of breast cancer. In the most recent decades breast cancer incidence has increased rapidly in many developing countries but only slowly in developed countries.^2^ In Thailand, the age-standardized incidence rate (ASR) of breast cancer is highest in Bangkok (34.1) and lowest in Nakhonphanom (13.1).^3^ As in other countries, breast cancer remains a public health problem for Thai women.

Study of the epidemiology and risk factors of breast cancer is very important for prevention. Hormonal contraceptive use is a risk factor for breast cancer, but the magnitude of the risk is unclear. Other possible risk factors include age,^4^ body mass index,^5^^,^^6^ family history of breast cancer,^7^ early menarche and late menopause,^8^ age at first childbirth,^9^ and shorter lifetime duration of breastfeeding in premenopausal women.^10^ A meta-analysis of case-control studies reported that oral hormonal contraceptive use increased the risk of premenopausal breast cancer.^11^

In 2005 the IARC reported that the global mean percentage of current use of oral contraception was 7.3% and that the rate was higher in developed (15.7%) than in developing (5.8%) regions. The mean rate for Southeast Asia was higher (12.8%) than that for Asia as a whole (4.5) but below that for the United States/Canada (15.5%) and Europe (17.4%). The rate for Thailand was surprisingly high (23%) and was similar to the mean for Australia/New Zealand (23.3%).^12^

It is unclear whether breast cancer risk differs according to the type of hormonal contraceptive used (oral, injection, or implant). One study found a high risk for a small group of women who had reported using implants.^13^

In this study we investigated the association between hormonal contraceptive use and breast cancer development in a Thai population. We expect that the findings will be valuable for breast cancer prevention and family planning programs.

## METHODS

This is an analytical study of data from a prospective cohort study, the Khon Kaen Cohort Study (KKCS), which is administered by the Cancer Unit of the Faculty of Medicine, Khon Kaen University, Thailand, and was established in 1991–2001. During this period, women aged 30 to 69 years living in Khon Kaen Province were invited to join the KKCS.^14^ Within the cohort there were 11 414 women with complete data on hormonal contraceptive use, and the analysis was done based on this number of participants. According to reports supplied by the Khon Kaen provincial cancer registry, there were 70 cases of histologically confirmed breast cancer among the 11 414 women. Follow-up was completed for all participants.

### Data collection

Variables of interest were age at recruitment, marital status, family history of breast cancer, breastfeeding history, age at first childbirth, age at menarche, and history of hormonal contraceptive use (including type of hormonal contraceptive used), and duration of use. Data for these variables were extracted from the KKCS dataset, which had been collected on recruitment into the cohort. All cohort participants were followed up until the end of the study, on December 31, 2011. Cohort participant data were linked to the Khon Kaen Cancer Registry to identify participants who had become breast cancer patients. The Khon Cancer Registry is the second population–based registry to be started in Thailand and was established in 1988 to represent the northeastern region of the country. Like the other 8 Thai cancer registries, it adheres to the procedures established by the IARC and International Association for Cancer Registration.^15^^,^^16^

All breast cancer diagnoses were histologically confirmed, and the date of diagnosis was obtained from medical records. Person-times were calculated from date of recruitment, date of diagnosis, date of loss to follow-up or withdrawal, and date at the end of the study.

### Statistical analysis

Multivariate analysis was used to investigate associations between hormonal contraceptive use and cancer development, and adjustments were made for age at recruitment, marital status, family history of breast cancer, and breastfeeding history. The Cox proportional hazards model was used to calculate risks of breast cancer in terms of hazard ratios (HRs) and 95% confidence intervals (CIs).

### Ethical approval

The study was conducted in accordance with the principles of the 1975 Declaration of Helsinki and was approved by the Khon Kaen University Ethics Committee for Human Research (Reference No. HE552223, date October 31, 2012).

## RESULTS

The study population of 11 414 provided a total observation time of 157 200 person-years; there were 70 cases of histologically confirmed breast cancer.

The demographic characteristics of the participants are summarized in Tables 1 and 2. To avoid the effects of confounding factors in the use of the Cox proportional hazard model, differences among various groups (never use and use of hormonal contraceptives, type of hormonal contraceptive used, duration of use, and age of first use) were adjusted for age at recruitment, marital status, family history of cancer, and breastfeeding history.

**Table 1.** Demographic characteristics of participants, by hormone use category (*n* = 11 414)

Variables	Never use of hormonal contraceptives *n* = 2153	Ever use										

		All contraceptive types *n* = 9261	Type of hormone contraceptive			Duration of use (years)			Age at first use (years)			
		
			Oral	Injected	Implant	<5	5–9	>9	<30	30–39	40–49	50^+^
			*n* = 5970	*n* = 3091	*n* = 200	*n* = 3664	*n* = 1802	*n* = 3795	*n* = 1252	*n* = 2813	*n* = 3252	*n* = 1944
1. Age at recruitment (years)												
30–39, *n* (%)	127 (5.9)	1390 (15.0)	970 (16.2)	327 (10.6)	93 (46.5)	538 (14.7)	320 (17.7)	532 (14.0)	758 (60.5)	632 (22.5)	0 (0.0)	0 (0.0)
40–49, *n* (%)	468 (21.7)	3662 (39.5)	2334 (39.1)	1248 (40.4)	80 (40.0)	1344 (36.7)	680 (37.7)	1638 (43.2)	472 (37.7)	1754 (62.5)	1436 (44.2)	0 (0.0)
50–59, *n* (%)	847 (39.3)	3195 (34.5)	1982 (33.2)	1190 (38.5)	23 (11.5)	1322 (36.1)	605 (33.6)	1268 (33.4)	21 (1.7)	421 (14.8)	1667 (51.3)	1086 (55.9)
60–69, *n* (%)	711 (33.1)	1014 (11.0)	684 (11.5)	326 (10.5)	4 (2.0)	460 (12.5)	197 (11.0)	357 (9.4)	1 (0.1)	6 (0.2)	149 (4.5)	858 (44.1)
2. Marital status												
Single/Divorce, *n* (%)	830 (38.6)	1304 (14.1)	878 (14.7)	409 (13.2)	17 (8.5)	545 (14.8)	247 (13.7)	512 (13.5)	55 (4.4)	271 (9.6)	464 (14.3)	514 (26.4)
Married, *n* (%)	1323 (61.4)	7957 (85.9)	5092 (85.3)	2682 (86.8)	183 (91.5)	3119 (85.2)	1555 (86.3)	3283 (86.5)	1197 (95.6)	2542 (90.4)	2788 (85.7)	1430 (73.6)
3. Family history of cancer												
No, *n* (%)	1480 (68.7)	6422 (69.3)	3973 (66.5)	2308 (74.7)	141 (70.5)	2795 (76.3)	1404 (77.9)	2223 (58.6)	641 (51.2)	1891 (67.2)	2368 (72.8)	1522 (78.3)
Yes, *n* (%)	673 (31.3)	2839 (30.7)	1997 (33.5)	783 (25.3)	59 (29.5)	869 (23.7)	398 (22.1)	1572 (41.4)	611 (48.8)	922 (32.8)	884 (27.2)	422 (21.7)
- Breast cancer, *n*	18	106	73	32	1	36	28	42	21	32	34	19
- Other cancer, *n*	540	2336	1568	714	54	785	307	1244	498	768	725	345
(Data on cancer type missing = 512)												

**Table 2.** Reproductive characteristics of participants, by hormone use category (*n* = 11 414)

Variables	Never use of hormone contraceptive *n* = 2153	Ever use										

		All contraceptive types *n* = 9261	Type of hormone contraceptive			Duration of use (years)			Age of first use (years)			
		
			Oral	Injected	Implant	<5	5–9	>9	<30	30–39	40–49	50^+^
			*n* = 5970	*n* = 3091	*n* = 200	*n* = 3664	*n* = 1802	*n* = 3795	*n* = 1252	*n* = 2813	*n* = 3252	*n* = 1944
Means (SD)												
1. Body mass index (kg/m^2^)	24.6 (3.8)	24.6 (3.8)	24.5 (3.8)	24.6 (3.8)	24.6 (3.5)	24.5 (3.8)	24.3 (3.8)	24.6 (3.7)	24.6 (3.5)	24.6 (3.8)	24.5 (3.8)	24.4 (3.8)
2. Age at menarche (years)	16.0 (1.6)	16.0 (1.8)	16.0 (1.6)	15.9 (1.6)	16.0 (1.6)	15.9 (1.7)	16.1 (1.8)	16.0 (1.7)	15.9 (1.8)	15.7 (1.7)	16.0 (1.7)	16.0 (1.7)
3. Age at menopause (years)	48.0 (7.2)	48.2 (7.4)	47.8 (7.4)	48.3 (7.2)	43.6 (6.8)	48.2 (7.4)	47.6 (7.4)	47.8 (7.2)	43.8 (6.7)	45.8 (7.2)	50.0 (6.2)	52 (6.4)
4. Age at first childbirth (years)												
<25, *n* (%)	1780 (82.7)	7624 (82.3)	4924 (82.5)	2543 (82.3)	157 (78.5)	3046 (83.1)	1463 (81.2)	3115 (82.1)	1088 (86.9)	226 (80.6)	2671 (82.1)	1597 (82.2)
25–29, *n* (%)	232 (10.8)	1202 (13.0)	766 (12.8)	401 (13.0)	35 (17.5)	456 (12.4)	270 (15.0)	476 (12.5)	138 (11.0)	412 (14.7)	390 (12.0)	262 (13.5)
>29, *n* (%)	141 (6.6)	435 (4.7)	280 (4.7)	147 (4.7)	8 (4.0)	162 (4.5)	69 (3.8)	204 (5.4)	26 (2.1)	133 (4.7)	191 (5.9)	85 (4.4)
5. Breastfeeding history												
Never, *n* (%)	313 (14.5)	159 (1.7)	131 (2.2)	24 (0.8)	4 (2.0)	56 (1.5)	36 (2.0)	67 (1.7)	17 (1.4)	60 (2.1)	53 (1.6)	29 (1.5)
Ever, *n* (%)	1840 (85.5)	9102 (98.3)	5839 (97.8)	3067 (99.2)	196 (98.0)	3608 (98.5)	1766 (98.0)	3728 (98.3)	1235 (98.6)	2753 (97.9)	3199 (98.4)	1915 (98.5)

The final results are shown in Table 3. Breast cancer risk among women with a history of hormonal contraceptive use was 1.31 times that of women without such a history, but this result was not statistically significant (95% CI, 0.65–2.65). In subgroup analysis, the HRs for breast cancer associated with hormonal contraceptive use were 0.86 (95% CI, 0.30–2.45) in women younger than 50 years and 1.91 (95% CI, 0.75–4.88) in women 50 years or older (Table 4).

**Table 3.** Association of hormone use with development of breast cancer

Factors	Number	Person-years	Number of breast cancer cases	Crude HR (95%CI)	Adjusted HR (95%CI)
1. Hormone use					
Never	2153	28 971.10	11	1.00	1.00
Ever	9261	128 229.50	59	1.34 (1.70–2.57)	1.31 (0.65–2.65)
2. Type of hormone					
Never	2153	28 971.10	11	1.00	1.00
Oral	5970	86 673.40	40	2.03 (0.94–4.35)	1.35 (0.65–2.78)
Injected	3091	38 402.90	18	1.80 (0.77–4.14)	1.25 (0.56–2.80)
Implant	200	3194	1	1.21 (0.15–9.77)	0.96 (0.12–7.73)
3. Duration of use (years)					
Never	2153	28 971.10	11	1.00	1.00
<5	3664	47 921.90	14	0.86 (0.39–1.92)	0.84 (0.36–1.94)
5–9	1802	25 485.80	17	2.10 (0.95–4.44)	1.95 (0.89–4.43)
>9	3795	54 821.70	28	1.45 (0.72–2.92)	1.45 (0.68–3.08)
				(*P* for trend = 0.08)	(*P* for trend = 0.09)
4. Age at first use (years)					
Never	2153	28 971.10	11	1.00	1.00
<30	1252	20 338.29	9	1.13 (0.47–2.74)	1.17 (0.36–3.73)
30–39	2813	40 629.15	18	1.29 (0.61–2.77)	1.07 (0.42–2.76)
40–49	3252	42 794.49	22	1.56 (0.75–3.26)	1.10 (0.49–2.50)
50^+^	1944	24 467.55	10	1.27 (0.53–3.02)	1.23 (0.45–4.26)
				(*P* for trend = 0.81)	(*P* for trend = 0.96)
5. Age at recruitment (years)					
30–39	1517	24 308.51	8	1.00	1.00
40–49	4130	58 230.55	28	1.58 (0.72–3.47)	1.59 (0.72–3.51)
50–59	4042	52 811.19	28	1.80 (0.81–3.97)	1.91 (0.86–4.28)
60–69	1725	21 850.34	6	0.93 (0.32–2.68)	1.14 (0.38–3.41)
				(*P* for trend = 0.26)	(*P* for trend = 0.43)
6. Marital status					
Single/Divorce	2134	28 221.61	8	1.00	1.00
Married	9280	128 978.98	62	1.68 (0.80–3.51)	1.79 (0.80–3.96)
7. Family history of cancer					
No	7902	102 683.51	47	1.00	1.00
Yes	3512	54 517.08	23	0.72 (0.42–1.24)	0.74 (0.43–1.29)
8. Breast feeding history					
Never	472	6883.43	4	1.00	1.00
Ever	10 942	150 317.16	66	0.76 (0.28–2.08)	0.49 (0.16–1.49)

Adjusted for age at recruitment, marital status, family history of cancer, and breastfeeding history.

**Table 4.** Association of hormone use with development of breast cancer: subgroup analysis stratified by age at start of follow-up

Hormone use	Numbers	Person-years	Number of breast cancer cases	Crude HR (95%CI)	^a^Adjusted HR (95%CI)
Women 30–49 years					
Never	595	9045.70	5	1.00	1.00
Ever	5052	73 493.36	31	0.85 (0.33–2.24)	0.86 (0.30–2.45)
Women 50–69 years					
Never	1558	19 925.41	6	1.00	1.00
Ever	4209	54 736.12	28	2.04 (0.82–5.06)	1.91 (0.75–4.88)

^a^Adjusted for marital status, family history of cancer, and breastfeeding history.

No type of hormonal contraceptive was associated with a significantly higher risk of breast cancer as compared with never use of hormonal contraceptives (oral contraception: HR = 1.35, 95% CI, 0.65–2.78; injection contraception: HR = 1.25, 95% CI, 0.56–2.80), and there was no relationship between duration of hormonal contraceptive use and breast cancer. Regarding age at beginning of follow-up, neither younger nor older women showed evidence of an association between hormonal contraceptive use and breast cancer risk.

## DISCUSSION

To our knowledge, this is the first study of the association between hormonal contraceptive use and breast cancer risk in Thai women. Our study did not find an association between hormonal contraceptive use and subsequent development of breast cancer. Similar results were reported in other studies. In a Norwegian study^17^ of 1423 women aged 40 to 60 years from families with hereditary familial breast cancer there were 380 cases of breast cancer, and ever use of oral contraceptives was unrelated to breast cancer risk (HR = 0.9; 95% CI, 0.68–1.18). However, among those who had last used this form of contraception 15 or more years previously, there was actually a reduced risk, and statistically significant increased risks were found for subgroups of more recent users (5–9 years and 10–14 years previously). A US population-based case-control study of 4575 women with invasive breast cancer and 4682 controls aged 35 to 64 years^18^ found that use of injectable progestin-only contraceptives was unrelated to breast cancer risk (OR = 0.9; 95% CI, 0.7–1.2). This absence of an association was found among current users (OR = 0.7; 95% CI, 0.4–1.3), those who began contraceptive use during the previous 5 years (OR = 0.9; 95% CI, 0.5–1.4), and those who began use before age 35 years (OR = 0.9; 95% CI, 0.6–1.3). Another study^19^ that used the same database found no association between breast cancer and oral contraceptive use among current or previous users (OR = 1.0; 95% CI, 0.8–1.3 and OR = 0.9; 95% CI, 0.8–1.0, respectively).

Other studies reported a positive association between hormonal contraceptive use and breast cancer risk. In a US study of 6150 female relatives of probands diagnosed with breast cancer during the period from 1944 to 1952, breast cancer developed in 242 relatives during the follow-up period. Ever use of oral contraceptives was associated with increased breast cancer risk in sisters and daughters (HR = 3.3; 95% CI, 1.6–6.7) but not in second-degree or no consanguineous relatives (HR = 1.2 for both). The authors noted that the increased risk among first-degree relatives was particularly evident among those who had used oral contraceptives before 1976, a period when they were likely to contain higher levels of estrogen and progestins. In a large prospective cohort study of 103 027 randomly selected Scandinavian women aged 20 to 49 years, 1008 women received a diagnosis of breast cancer during the duration of follow-up. Ever-use was associated with increased risk (HR = 1.3; 95% CI, 1.1–1.5, adjusted for age, parity, age at first birth, and 8 other covariates, including family history).

The present lack of an association of breast cancer with oral and injected contraceptive use is similar to findings from other studies.^13^^,^^20^ The absence of a statistically significant linear relationship between duration of hormonal contraceptive use and breast cancer risk was also reported by others.^21^^–^^23^ However, 1 study found a weak, but statistically significant, trend toward increased risk with longer duration of use,^24^ and a Canadian study reported a significant inverse association.^25^

The lack of consistency in past and present findings is difficult to explain but is probably due to differences between the populations studied, such as recency of use, family history, and number of cases. Furthermore, the data on hormonal contraceptive use in our study came from a single interview at cohort recruitment. This might be a limitation, because some women may have started using hormonal contraceptives after baseline, and a subgroup analysis showed that HRs for hormone use differed between younger and older women. The statistical power of the test was calculated based on the Cox proportional hazards model with an expected HR of 1.5, but the small number of breast cancer cases (*n* = 70) limited the power of this study.

In conclusion, we found no association between hormonal contraceptive use and breast cancer; however, this finding should be viewed with caution due to the small number of cases.

## References

[1] Ferlay J, Shin HR, Bray F, Forman D, Mathers C, Parkin DM Estimates of worldwide burden of cancer in 2008: GLOBOCAN 2008. Int J Cancer. 2010;127:2893–917 10.1002/ijc.25516 21351269

[2] International Agency for Research on Cancer. Cancer Incidence, Mortality and Prevalence Worldwide in 2008 [homepage on the Internet]. Lyon: International Agency for Research on Cancer; c2013 [cited 2012 May 22]. Available from: http://www.iarc.fr/en/publications/pdfs-online/wcr/2008/wcr_2008.pdf

[3] Khuhaprema T, Srivatanakul P, Attasara P, Sriplung H, Wiangnon S, Sumitsawan Y, editors. Cancer in Thailand Vol. V, 2001–2003. Bangkok: Bangkok Medical Publisher; 2010.

[4] Ewertz M, Mellemkjaer L, Poulsen AH, Friis S, Sørensen HT, Pedersen L, Hormone use for menopausal symptoms and risk of breast cancer. A Danish cohort study. Br J Cancer. 2005;92:1293–7 10.1038/sj.bjc.6602472 15785751 PMC2361963

[5] Eichholzer M, Schmid SM, Bovey F, Jordan P, Rohrmann S, Huang DJ, Impact of overweight and obesity on postmenopausal breast cancer: analysis of 20-year data from Switzerland. Arch Gynecol Obstet. 2012;285:797–803 10.1007/s00404-011-2022-7 21814854

[6] Teras LR, Goodman M, Patel AV, Diver WR, Flanders WD, Feigelson HS Weight loss and postmenopausal breast cancer in a prospective cohort of overweight and obese US women. Cancer Causes Control. 2011;22:573–9 10.1007/s10552-011-9730-y 21327461

[7] Phipps AI, Chlebowski RT, Prentice R, McTiernan A, Wactawski-Wende J, Kuller LH, Reproductive history and oral contraceptive use in relation to risk of triple-negative breast cancer. J Natl Cancer Inst. 2011;103:470–7 10.1093/jnci/djr030 21346227 PMC3057984

[8] Shin A, Song YM, Yoo KY, Sung J Menstrual factors and cancer risk among Korean women. Int J Epidemiol. 2011;40:1261–8 10.1093/ije/dyr121 21841186

[9] Matalqah L, Radaideh K, Yusoff ZM, Awaisu A Predictors of breast cancer among women in a northern state of Malaysia: a matched case-control study. Asian Pac J Cancer Prev. 2011;12:1549–53 22126497

[10] Gajalakshmi V, Mathew A, Brennan P, Rajan B, Kanimozhi VC, Mathews A, Breastfeeding and breast cancer risk in India: a multicenter case-control study. Int J Cancer. 2009;125:662–5 10.1002/ijc.24429 19452516

[11] Kahlenborn C, Modugno F, Potter DM, Severs WB Oral contraceptive use as a risk factor for premenopausal breast cancer: a meta-analysis. Mayo Clin Proc. 2006;81:1290–302 10.4065/81.10.1290 17036554

[12] IARC Working Group on the Evaluation of Carcinogenic Risks to Humans Combined Estrogen-progestogen Contraceptives and combined estrogen-progestogen menopausal therapy. IARC Monograph, Volume 91. WHO, France; 2007. PMC4781221 18756632

[13] Sweeney C, Giuliano AR, Baumgartner KB, Byers T, Herrick JS, Edwards SL, Oral, injected and implanted contraceptives and breast cancer risk among U.S. Hispanic and non-Hispanic white women. Int J Cancer. 2007;121:2517–23 10.1002/ijc.22970 17657739

[14] Sriamporn S, Parkin DM, Pisani P, Vatanasapt V, Suwanrungruang K, Kamsa-ard P, A prospective study of diet, lifestyle, and genetic factors and the risk of cancer in Khon Kaen Province, northeast Thailand: description of the cohort. Asian Pac J Cancer Prev. 2005;6:295–303 16235989

[15] Suwanrungruang K, Sriplung H, Attasara P, Temiyasathit S, Buasom R, Waisri N, Quality of case ascertainment in cancer registries: a proposal for a virtual three-source capture-recapture technique. Asian Pac J Cancer Prev. 2011;12:173–8 21517253

[16] Suwanrungruang K, Sriplung H, Temiyasathit S, Waisri N, Daoprasert K, Kamsa-Ard S, Appropriateness of the standard mortality/incidence ratio in evaluation of completeness of population-based cancer registry data. Asian Pac J Cancer Prev. 2011;12:3283–8 22471467

[17] Heimdal K, Skovlund E, Møller P Oral contraceptives and risk of familial breast cancer. Cancer Detect Prev. 2002;26:23–7 10.1016/S0361-090X(02)00004-1 12088199

[18] Strom BL, Berlin JA, Weber AL, Norman SA, Bernstein L, Burkman RT, Absence of an effect of injectable and implantable progestin-only contraceptives on subsequent risk of breast cancer. Contraception. 2004;69:353–60 10.1016/j.contraception.2003.12.015 15105056

[19] Marchbanks PA, McDonald JA, Wilson HG, Folger SG, Mandel MG, Daling JR, Oral contraceptives and the risk of breast cancer. N Engl J Med. 2002;346:2025–32 10.1056/NEJMoa013202 12087137

[20] Grabrick DM, Hartmann LC, Cerhan JR, Vierkant RA, Therneau TM, Vachon CM, Risk of breast cancer with oral contraceptive use in women with a family history of breast cancer. JAMA. 2000;284:1791–8 10.1001/jama.284.14.1791 11025831

[21] Kumle M, Weiderpass E, Braaten T, Persson I, Adami HO, Lund E Use of oral contraceptives and breast cancer risk: The Norwegian-Swedish Women’s Lifestyle and Health Cohort Study. Cancer Epidemiol Biomarkers Prev. 2002;11:1375–81 12433714

[22] Urban M, Banks E, Egger S, Canfell K, O’Connell D, Beral V, Injectable and oral contraceptive use and cancers of the breast, cervix, ovary, and endometrium in black South African women: case-control study. PLoS Med. 2012;9:e1001182 10.1371/journal.pmed.1001182 22412354 PMC3295825

[23] Rohan TE, Miller AB A cohort study of oral contraceptive use and risk of benign breast disease. Int J Cancer. 1999;82:191–6 10.1002/(SICI)1097-0215(19990719)82:2<191::AID-IJC7>3.0.CO;2-F 10389751

[24] Shapiro S, Rosenberg L, Hoffman M, Truter H, Cooper D, Rao S, Risk of breast cancer in relation to the use of injectable progestogen contraceptives and combined estrogen/progestogen contraceptives. Am J Epidemiol. 2000;151:396–403 10.1093/oxfordjournals.aje.a010219 10695598

[25] Silvera SA, Miller AB, Rohan TE Oral contraceptive use and risk of breast cancer among women with a family history of breast cancer: a prospective cohort study. Cancer Causes Control. 2005;16:1059–63 10.1007/s10552-005-0343-1 16184471

